# Knowledge map of artificial intelligence in neurodegenerative diseases: a decade-long bibliometric and visualization study

**DOI:** 10.3389/fnagi.2025.1586282

**Published:** 2025-05-14

**Authors:** Junwei Huang, Shuqi Wang, Xuankai Liao, Danting Su, Rubing Lin, Tao Zhang, Long Zhao

**Affiliations:** ^1^Sydney Smart Technology College, Northeastern University at Qinhuangdao, Qinhuangdao, China; ^2^Department of Clinical Laboratory, Affiliated Hospital of North Sichuan Medical College, Nanchong, China; ^3^Department of Pathology, The Seventh Affiliated Hospital, Sun Yat-Sen University, Shenzhen, China; ^4^Department of Orthopedics, Shenzhen Children’s Hospital, Shenzhen, China; ^5^Department of Neurosurgery, Affiliated Hospital of North Sichuan Medical College, Nanchong, China

**Keywords:** artificial intelligence, neurodegenerative diseases, Parkinson’s disease, bibliometrics, Web of Science

## Abstract

**Background:**

As the incidence of neurodegenerative diseases increases, the related AI research is getting more and more advanced. In this study, we analyze the literature in this field over the last decade through bibliometric and visualization methods with the aim of mining the prominent journals, institutions, authors, and countries in this field and analyzing the keywords in order to speculate on possible future research trends.

**Methods:**

Our study extracted 1,921 relevant publications spanning 2015–2025 from the Web of Science Core Collection database. We conducted comprehensive bibliometric analyses and knowledge mapping visualizations using established scientometric tools: CiteSpace and Bibliometrix.

**Results:**

A total of 1921 documents were included in the study, the number of publications in this field showed an overall increasing trend, and the average number of citations showed a downward trend since 2019. Among the journals, *Scientific Reports* had the highest number of publications. In addition, we identified 22 core journals. Institution wise, University of London has the highest participation. Among the authors, the highest number of publications is Benzinger, Tammie. The highest number of citations is Fingere Elizabeth. At the national level, the United States is number one in the world in terms of influence in this field, and China is ranked number two, both of which are well ahead of other countries and are major contributors to this field. The analysis of keywords showed the centrality of Alzheimer disease, machine learning, Parkinsons disease, and deep learning. All the studies were clustered based on keywords to get seven clusters: 0. immune infiltration; 1. Parkinsons disease; 2. multiple sclerosis; 3. mild cognitive impairment; 4. deep learning; 5. machine learning; 6. freesurfer; 7. scale. In addition, we also found the continuation of the trending topics, which are Parkinsons disease, deep learning, and machine learning.

**Conclusion:**

Based on the relationship between keywords and time, we speculate that there are four possible research trends: 1. Precision diagnosis with multimodal data fusion. 2. Pathological mechanism analysis and target discovery. 3. Interpretable AI and clinical translation. 4. Technology differentiation for subdivided diseases.

## 1 Introduction

Neurodegenerative diseases (e.g., Parkinson’s disease, Alzheimer’s disease, Huntington’s disease, etc.) are a major challenge to global public health, with highly complex pathomechanisms (Li et al., 2024), highly age-related morbidity, and a tendency to spread rapidly due to increasing population aging. In China, the number of Alzheimer’s disease patients among people over 60 years old has reached 9.83 million cases, while Parkinson’s disease patients are expected to reach 5 million by 2030, accounting for nearly half of the world (Ren et al., 2022).

The chronic and progressive nature of these diseases makes early diagnosis difficult. Currently, diagnosis mainly relies on clinical symptom observation and imaging tests (e.g., MRI, PET scans), but early non-specific symptoms (e.g., decreased sense of smell, sleep disorders) are easily overlooked, and misdiagnosis rates are high (Tolosa et al., 2021). For example, the diagnosis of Parkinson’s disease needs to be combined with symptoms of dyskinesia, while pathological markers [e.g., α-synuclein (α-Syn) abnormalities (Hatano et al., 2024)] can only be confirmed by autopsy, and there is a lack of specific tests in the clinic. Although emerging technologies such as the novel PET tracer “[18F]-F0502B” have successfully visualized α-synuclein in mouse models, its clinical application is still in the experimental stage (Xiang et al., 2023).

Artificial intelligence (AI) technology has been developing rapidly in recent years, and many fields have benefited from new breakthroughs in the development of AI (LeCun et al., 2015; Wang et al., 2023). AI has also brought new hope in the diagnosis, prediction and treatment of neurodegenerative diseases (Wang et al., 2023; Li et al., 2022; Li et al., 2023; Wang et al., 2023). Especially in the field of machine learning and deep learning, AI technology is able to extract key information in complex medical data through big data analysis and pattern recognition, which in turn helps doctors achieve early diagnosis, accurate prediction and personalized treatment (Kermany et al., 2018). For example, convolutional neural networks in deep learning have been successfully applied to medical image analysis, and through automated analysis of MRI and CT scan images of the brain, AI is able to identify small changes in the brain related to neurodegenerative diseases (Jia et al., 2024). Such technologies have not only increased diagnostic efficiency, but also significantly improved the accuracy of early diagnosis of diseases. Not only that, but AI has also shown great potential in genomics and biomarker discovery (Shin et al., 2016). Through in-depth analysis of patients’ genomic data, AI is able to reveal genetic variants and potential biomarkers associated with neurodegenerative diseases, which also provides a theoretical basis for precision medicine and personalized treatment (De Fauw et al., 2018). For example, deep learning models have been able to extract genetic markers important for the early diagnosis of Parkinson’s disease or Alzheimer’s disease from massive amounts of genetic data, enabling the customization of individualized treatment plans (Zhou et al., 2023).

Globally, several countries and organizations have begun to apply AI to the research and clinical practice of neurodegenerative diseases. Researchers at Google DeepMind have developed an AI-based diagnostic system for fundus disorders, which has achieved significant clinical results (Powles and Hodson, 2017). These studies are driving the development of AI technology in medicine, and there is no doubt that AI is accelerating toward the clinic.

Bibliometric studies can analyze existing literature from multiple perspectives, presenting a rich knowledge graph for the target field (Ninkov et al., 2022; Tu et al., 2024). Bibliometric analysis can also work with experimental analysis to explore the target area together, making up for the shortcomings of using bibliometric analysis alone, and this method may become the mainstream method in the future (Kumar and Azad, 2024).

Neurodegenerative diseases are also rapidly evolving with the advancement of AI, and the plethora of information may overwhelm researchers, and the existing studies do not present a more comprehensive knowledge map for researchers. So, a time-sensitive, comprehensive bibliometric study is just what is needed under this field.

The purpose of this study is to dig out the prominent sources, authors, institutions and countries under the field and discuss their background. To analyze the keywords and find out the research hotspots over the years and predict the possible research trends in the coming years.

## 2 Methods

### 2.1 Data sources and retrieval methods

**Figure 1:**
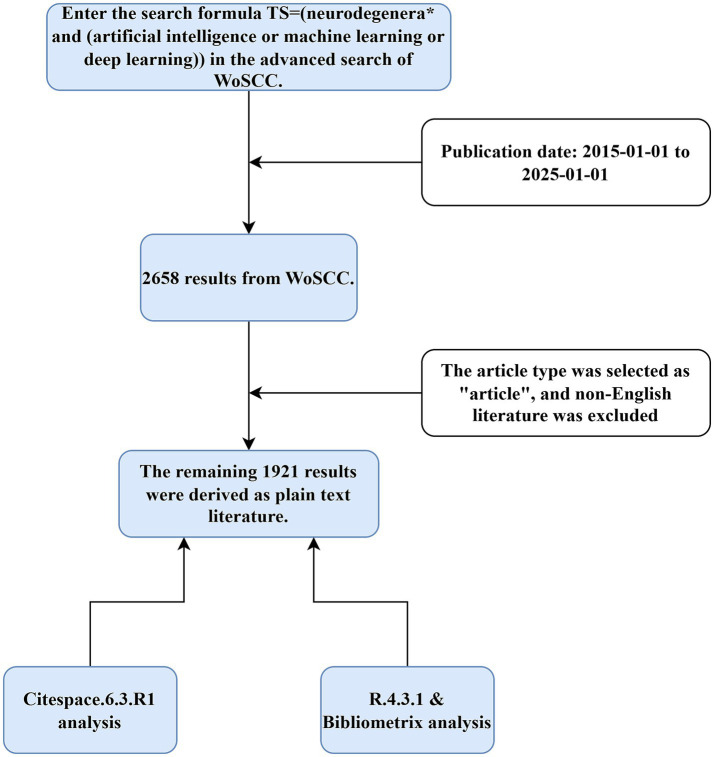
Flow chart: the main processes are target literature search, screening and processing using two software programs.

The literature collected for this research is all from Web of Science Core Collection (WoSCC). WoSCC has strict income criteria, collecting literature from this database can avoid collecting too much literature with low research value thus ensuring the accuracy of this research. After discussion, we determined the search formula TS = [neurodegenera* and (artificial intelligence or machine learning or deep learning)], entered the search formula and the time range was set from January 1, 2015, to January 1, 2025, the search results contain 2,658 articles, refined articles and English literature, got 1,921 articles, these documents will be exported in the form of plain text with complete records and citations, got the original data we need. Figure 1 shows the general flow of this study.

### 2.2 Procedure operation

The tools utilized in this research are CiteSpace and the bibliometrix package for R language.

The collected plain text data is imported into cite space and duplicates are first removed. After removing the duplicates, create a new project, select the time interval from January 2015 to January 2025, select the time slice as 1, and leave the rest of the settings as default. Then in the “Node Types” single-selected items for visual analysis.

Compress the original file into a zip file and import it into bibliometrix, run it under each category to get the corresponding results.

Detailed instructions can be found in the respective manuals.

## 3 Results

### 3.1 Overview

**Figure 2:**
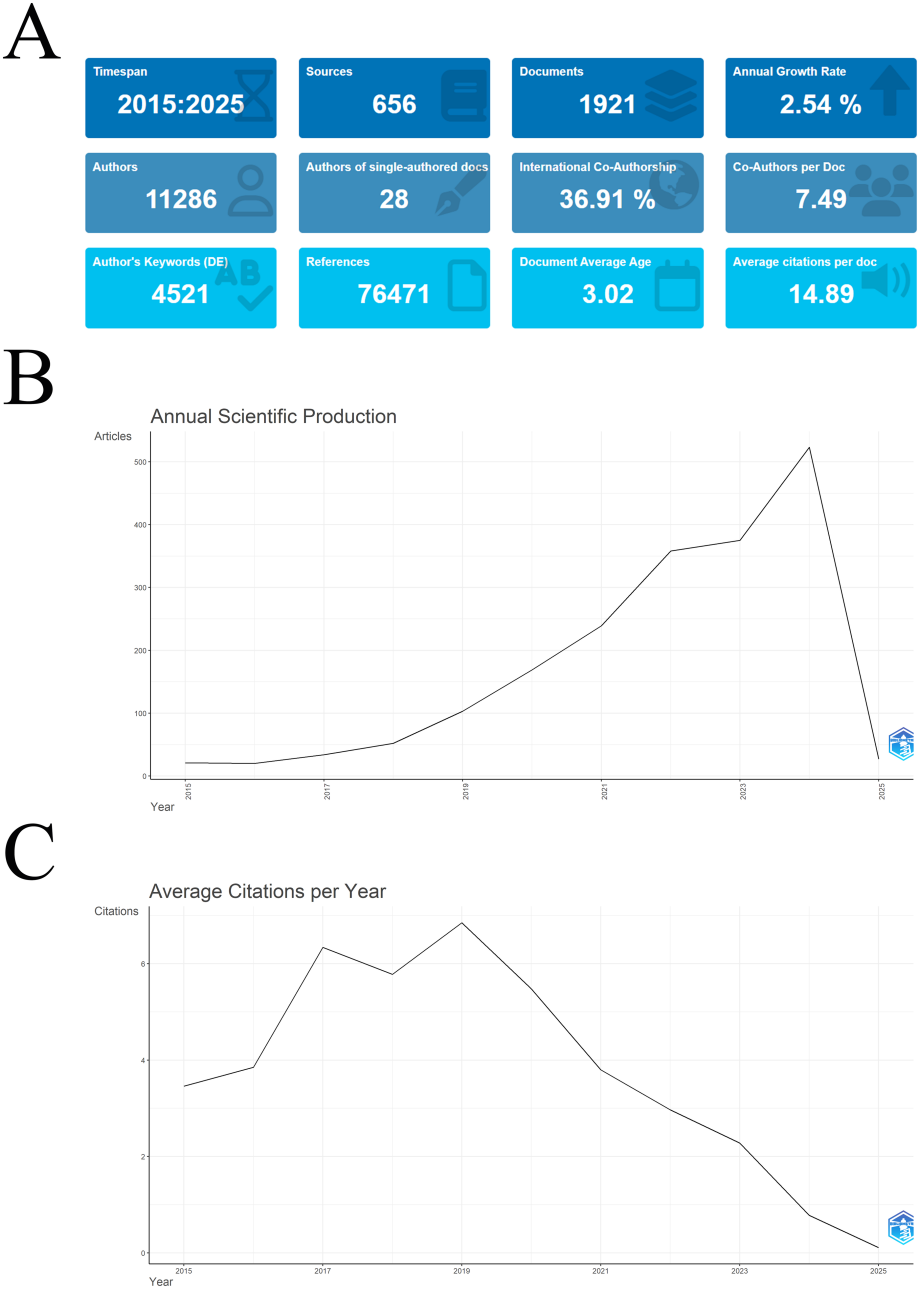
**(A)** The figure contains 12 main information about the target literature such as Timespan, Sources etc.; **(B)** This graph reflects the new releases per year since 2015, with a general upward trend. Note that the time interval was chosen to be January 2025, but subsequent studies were not included, so the curve shows a rapid downward shift; **(C)** This graph shows the average annual citation frequency of the target literature. As can be seen, using 2019 as the cutoff, there is a general upward trend until that point, followed by a yearly decline.

All the literature spans a period of 10 years (2015–2025), totaling 1,921 documents, which originated from 656 journals, with a total of 11,286 authors contributing to the writing, with an average of 7.49 authors contributing to each document. The average age of the literature is 3.02, indicating that most of these documents were published in recent years, and more information is shown in Figure 2A. The scientific output per year under this field is illustrated in Figure 2B, which shows a positive growth per year excluding 2025, which has just started. Figure 2C, on the other hand, shows the average citations per year for these literatures. We can clearly see an overall increasing trend in the average number of citations of articles until 2019, but after that, the curve declines rapidly and the average number of citations per year falls rapidly. Looking at Figure 2B again based on this phenomenon, we find that 2019 can be used as a dividing line, with the literature growing slowly year by year before this year and increasing rapidly after this year. We speculate that there may be too many articles with low citation counts published after 2019, and these articles lower the annual average citation count.

### 3.2 Keywords

**Figure 3:**
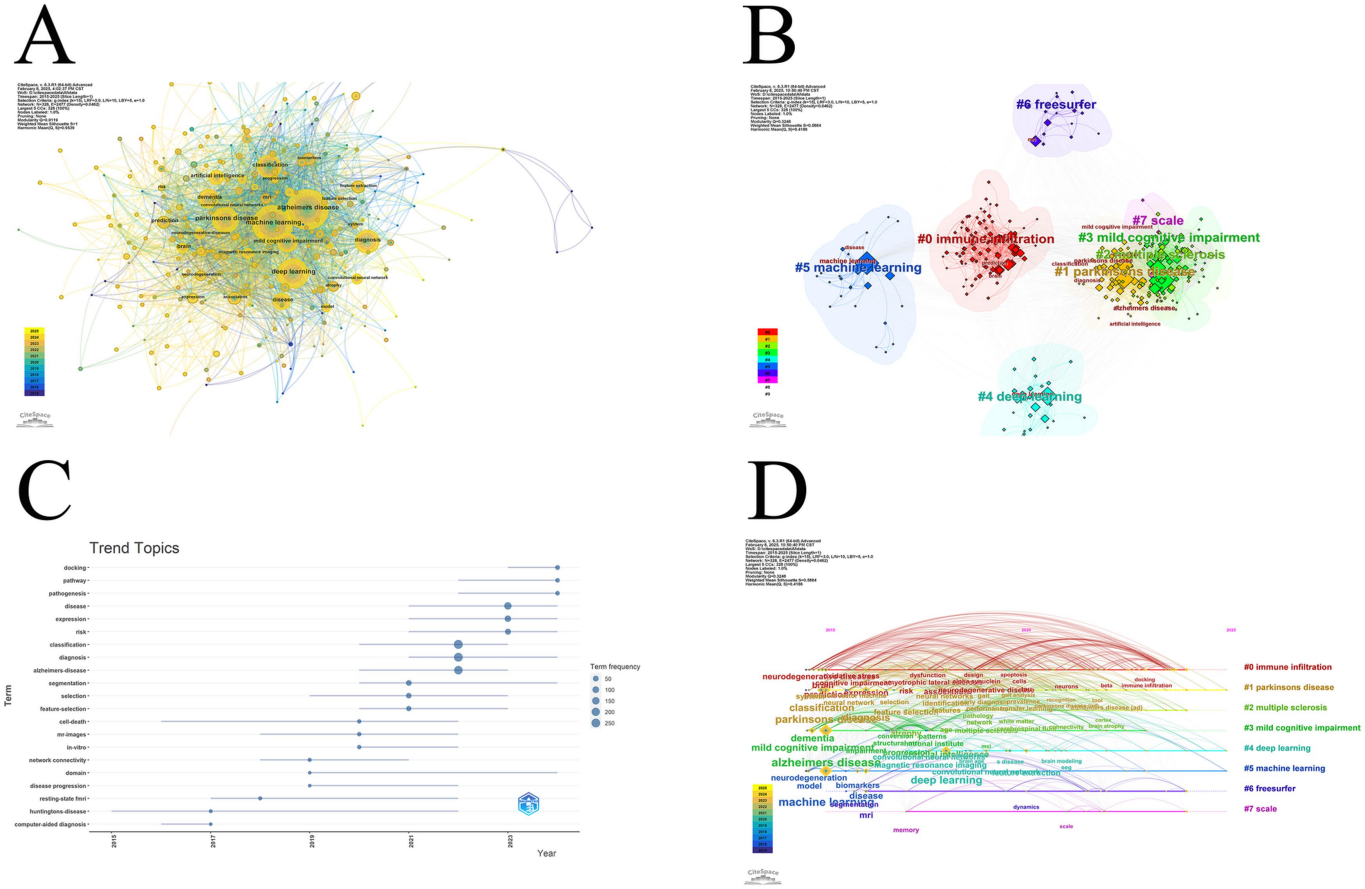
**(A)** Co-occurrence network of keywords in the target literature. The larger the node and the closer the position to the center, the stronger the relevance of this keyword. E.g., machine learning, Alzheimer disease, Parkinsons disease, etc.; **(B)** The target literature is clustered according to keywords. There are eight clusters, four of which are more closely related, indicating that they often occur together; **(C)** Trending topics reflect the evolution of popular topics over the selected time interval, and several topics that persist to this day such as docking, pathway, pathogenesis, etc. suggest future research trends; **(D)** This graph shows the keyword clustering results in the form of a timeline, looking at the distribution of keywords in each cluster over time.

For keyword analysis, we first obtained the keyword co-occurrence network graph (Figure 3A). The larger nodes in the graph are Alzheimer disease, machine learning, Parkinsons disease, and deep learning. It shows that these keywords appear more frequently in this field. Based on the colors we can also see that these keywords are still active in recent years. Based on the keywords, eight clusters can be obtained by categorizing these literatures:

0 immune infiltration
1 Parkinsons disease
2 multiple sclerosis
3 mild cognitive impairment
4 deep learning
5 machine learning
6 freesurfer
7 scale

Four of the seven clusters, Parkinsons disease, multiple sclerosis, mild cognitive impairment, and scale, are more closely linked, indicating that these four topics often appear together (Figure 3B). In trend topics (Figure 3C) one can see the topics that have emerged during the selected time interval. The earliest start was research on hentingtons disease, which continues until the end of 2022. There are more papers on classification, diagnosis and Alzheimer disease, with the diagnosis theme continuing to the present day. Other themes that continue to this day are docking, pathway, pathogenesis, disease, expression, risk, segmentation, domain. There is a similar situation in the clusters just categorized, with three clusters continuing to this day, Parkinsons disease, deep learning and machine learning. Deep learning and machine learning (Figure 3D).

### 3.3 Source

**Figure 4:**
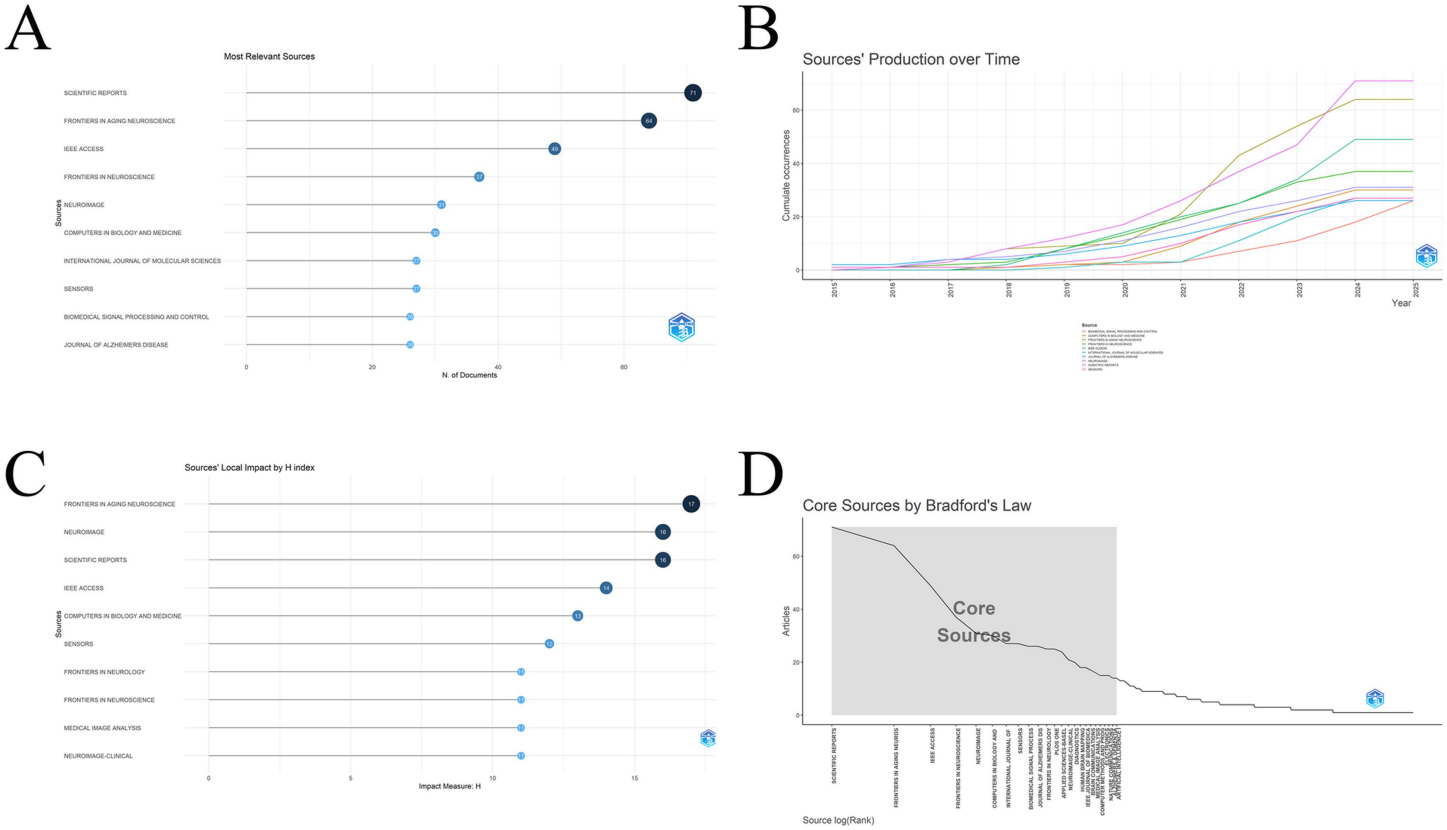
**(A)** The figure shows the top 10 most relevant journals, which are listed in order of the number of target articles published. Seventy-one of these articles were published by *Scientific Report*, which is why it is in first place; **(B)** This graph shows the number of publications per year for the top 10 most relevant journals, and the overall trend can be categorized into slow-growth and fast-growth periods. The graph also shows the start time of each journal’s acceptance of the target journals, e.g., *Biomedical Signal Processing and Control*, *IEEE Access*, etc. started accepting them only after 2017; **(C)** This chart shows the top 10 journals ranked according to the H index, with seven journals having appeared in the previous list at the same time: Frontiers in Aging Neuroscience, Neuroimage, Scientific Reports, IEEE Access, Computer in Biology and Medicine, Sensor, Frontiers in Neuroscience; **(D)** Distribution curve of core journals.

Of the 656 journal sources *Scientific Reports* published the most articles, followed by *Frontiers in Aging Neuroscience, IEEE Access, Frontiers in Neuroscience*, and *Neuroimage*. the top 10 journals in terms of number of articles published are presented in Figure 4A and the specific number. The year-by-year publication volume of these journals is shown in Figure 4B. Four of these journals hardly accepted articles in this field in the early years, which are *Biomedical Signal Processing and Control*, *Computers in Biology and Medicine, IEEE Access*, and *International Journal of Molecular Science*. these four journals started late but started receiving them one by one after 2017 and are still among the top 10. The first journals to start receiving articles in this area were *Journal of Alzheimers Disease* and *Scientific Reports*. they started receiving before 2015, which shows the foresight of the editors-in-chief of these two journals. We then examined the h-index of each journal, and the results are shown in Figure 4C. the highest h-index was 17, from *Frontiers in Aging Neuroscience.* this was followed by 16 from two journals, *Neuroimage* and *Scientific Reports*, which tied for second place. The h-index takes into account the number of publications and the number of citations, and can provide a measure of the level of the journal. It can assess the level of journals to some extent. Table 1 contains the h_index, g_index, m_index, total citations (TC), number of papers (NP), and year of start (PY_start) for the top 10 journals in the h-index. Based on Bradford’s law, we identified the core journals under this field, including *Scientific Reports*, *Frontiers in Aging Neuroscience*, and *IEEE Access* (Figure 4D). The remaining core journals are listed in Table 2.

**Table 1:** The top 10 journals of the H-index.

Element	h_index	g_index	m_index	TC	NP	PY_start
FRONTIERS IN AGING NEUROSCIENCE	17	27	1.7	900	64	2016
NEUROIMAGE	16	31	1.6	1,296	31	2016
SCIENTIFIC REPORTS	16	34	1.455	1,241	71	2015
IEEE ACCESS	14	29	1.75	893	49	2018
COMPUTERS IN BIOLOGY AND MEDICINE	13	27	1.625	740	30	2018
SENSORS	12	21	1.2	448	27	2016
FRONTIERS IN NEUROLOGY	11	18	1.375	336	25	2018
FRONTIERS IN NEUROSCIENCE	11	24	1	615	37	2015
MEDICAL IMAGE ANALYSIS	11	16	1.222	521	16	2017
NEUROIMAGE-CLINICAL	11	20	1	408	21	2015

**Table 2:** Core journals in the field.

SO	Rank	Freq	cumFreq	Zone
SCIENTIFIC REPORTS	1	71	71	Zone 1
FRONTIERS IN AGING NEUROSCIENCE	2	64	135	Zone 1
IEEE ACCESS	3	49	184	Zone 1
FRONTIERS IN NEUROSCIENCE	4	37	221	Zone 1
NEUROIMAGE	5	31	252	Zone 1
COMPUTERS IN BIOLOGY AND MEDICINE	6	30	282	Zone 1
INTERNATIONAL JOURNAL OF MOLECULAR SCIENCES	7	27	309	Zone 1
SENSORS	8	27	336	Zone 1
BIOMEDICAL SIGNAL PROCESSING AND CONTROL	9	26	362	Zone 1
JOURNAL OF ALZHEIMERS DISEASE	10	26	388	Zone 1
FRONTIERS IN NEUROLOGY	11	25	413	Zone 1
PLOS ONE	12	25	438	Zone 1
APPLIED SCIENCES-BASEL	13	24	462	Zone 1
NEUROIMAGE-CLINICAL	14	21	483	Zone 1
DIAGNOSTICS	15	20	503	Zone 1
HUMAN BRAIN MAPPING	16	18	521	Zone 1
IEEE JOURNAL OF BIOMEDICAL AND HEALTH INFORMATICS	17	18	539	Zone 1
BRAIN COMMUNICATIONS	18	17	556	Zone 1
MEDICAL IMAGE ANALYSIS	19	16	572	Zone 1
COMPUTER METHODS AND PROGRAMS IN BIOMEDICINE	20	15	587	Zone 1
ELECTRONICS	21	15	602	Zone 1
NATURE COMMUNICATIONS	22	15	617	Zone 1
ALZHEIMERS & DEMENTIA	23	14	631	Zone 1
ARTIFICIAL INTELLIGENCE IN MEDICINE	24	14	645	Zone 1
BRAIN	25	13	658	Zone 2

### 3.4 Author

**Figure 5:**
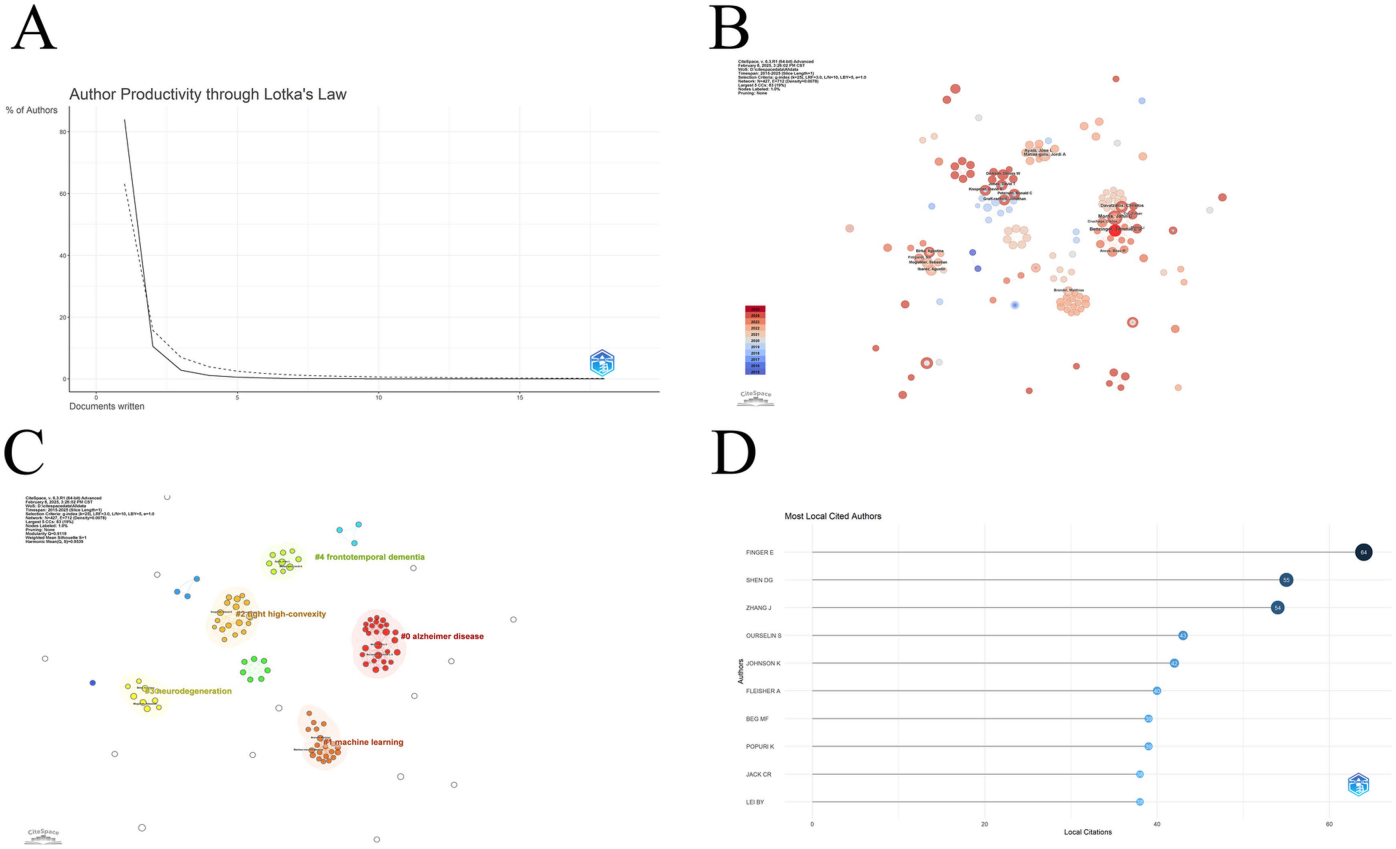
**(A)** Author distribution curve according to the number of publications. The curve shows that most authors in this field have published only one or two articles; **(B)** Like the Institutional Collaboration Network, this diagram shows the Author Collaboration Network; **(C)** The author based on the keyword clustering results. There are roughly five main groups, and there is closer cooperation within the groups than between them; **(D)** The figure shows the top 10 authors in terms of local citations. This ranking reflects the influence of the authors in the target field.

In 1926, the American mathematician Alfred Lotka proposed Lotka’s Law to describe the distribution of literature productivity. Lotka’s law, which describes the distribution of productivity in the literature, states that the number of outputs of researchers in a given field of study follows a certain pattern, with about 60% of authors publishing only one paper. About 15% of authors publish two papers, which is a quarter of the number of authors who publish one paper. About 6.7% of authors publish three papers, which is one-ninth of the number of authors who publish one paper. The dashed line in Figure 5A shows the standard distribution curve, and the realization shows the distribution curve under this domain. It is clear that the overall picture is consistent with Lotka’s law. However, the percentage of authors with only one publication exceeds 80%. This indicates that there are very few highly productive authors in this field and that the field is in a rapid development phase. A similar picture can be seen through the author co-occurrence network (Figure 5B). The size of each node represents the number of collaborations as well as the number of publications, and there is no great disparity in the size of the nodes in the figure, with the more prominent being Benzinger, Tammie (Washington University), an author who has been more active in recent research. The colors correspond to the years in the bottom left corner, and the colors suggest that most of the authors are also active after 2020. Based on this partnership, we performed a cluster analysis of these authors, and the results are shown in Figure 5C. Most of the authors can be categorized into five clusters around the five topics of frontotemporal dementia, Alzheimer disease, machine learning, tight high-convexity, and neurodegeneration research. Among these authors, Finger Elizabeth (University of Western Ontario) has the highest number of citations, followed by Shen Dinggang (ShanghaiTech University), and Zhang Jian (Shanghai Jiao Tong University) in the third place. Among these authors, Finger Elizabeth (University of Western Ontario) has the highest number of citations, followed by Shen Dinggang (ShanghaiTech University), and Zhang Jian (Shanghai Jiao Tong University) in the third place (Figure 5D). Table 3 lists the top seven authors in terms of H_index, the first being Ibanez, Agustin (University of California San Francisco), the second being Jack, Clifford R (Mayo Clinic). Third is Moguilner, Sebastian (University of San Francisco).

**Table 3:** The top seven authors of the H-index.

Element	h_index	g_index	m_index	TC	NP	PY_start
IBÁÑEZ A	8	8	1	181	8	2018
JACK CR	8	9	1	429	9	2018
MOGUILNER S	8	9	1	192	9	2018
AYALA JL	7	9	0.875	153	9	2018
BIRBA A	7	7	1.4	129	7	2021
LIU Y	7	13	0.778	177	18	2017
PETERSEN RC	7	10	0.875	218	10	2018

### 3.5 Organization

**Figure 6:**
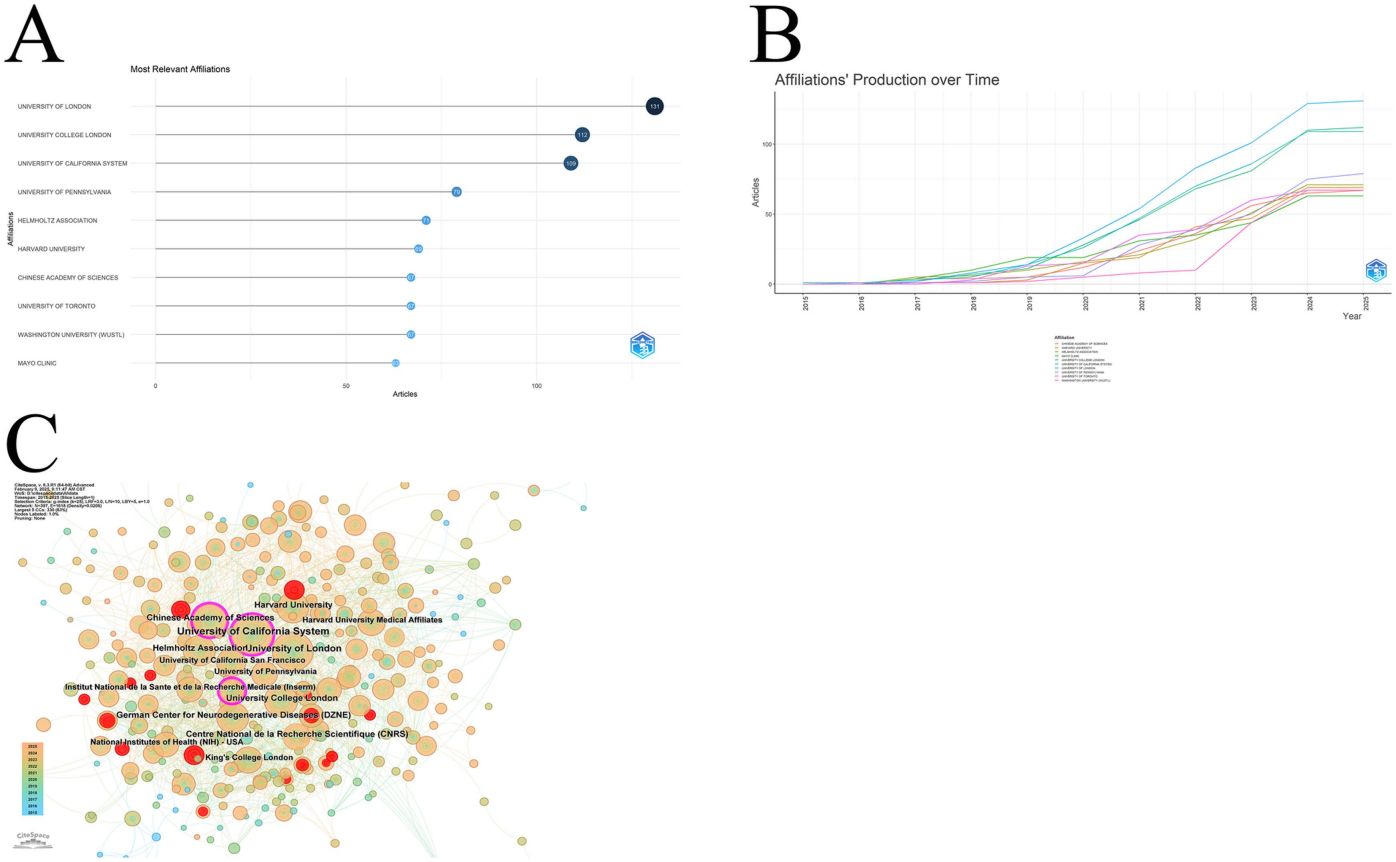
**(A)** The graph shows the top 10 most relevant institutions, again in order of the number of target articles published; **(B)** This graph shows the volume of publications by year for the 10 most relevant institutions, which can likewise be divided into slow-growth and fast-growth periods; **(C)** The figure shows the collaboration network of the institution, where larger nodes indicate more collaborations, and different colors correspond to the years in the lower left corner. A red node indicates that there is a burst of publication at this institution.

Analyzing the institutions to which this literature belongs, we obtained the following results: university of London had the highest participation, with 131 articles involving researchers from this institution. The second place is university college London with 112 articles. The third place is university of California system with 109 articles of participation. The remaining top 10 institutions are presented in Figure 6A. Most of these institutions started their research in this area in 2016, and only two institutions started publishing papers in 2015, which are university of California system and University of Pennsylvania. of these institutions, university of California system researched this area earlier than 2015. The overall trend is the same as the trend in this field, with 2018 to 2019 being a transition year, and the number of published articles increasing rapidly after 2019 (Figure 6B). The collaborations in each institution are shown in the Institutional Contribution Network, where a larger node in the graph indicates that it collaborates more frequently with other institutions, with different colored year rings representing different times as shown in the lower left corner. The most frequent collaboration is with the university of California system, followed by the university of London and university college London. red nodes in the graph are institutions that have been more active in recent years, although their current number of collaborations is low, but basically concentrated in recent years. For example, University of Chinese Academy of Sciences, Capital Medical University, University of Munich, etc. (Figure 6C).

### 3.6 Country and region

**Figure 7:**
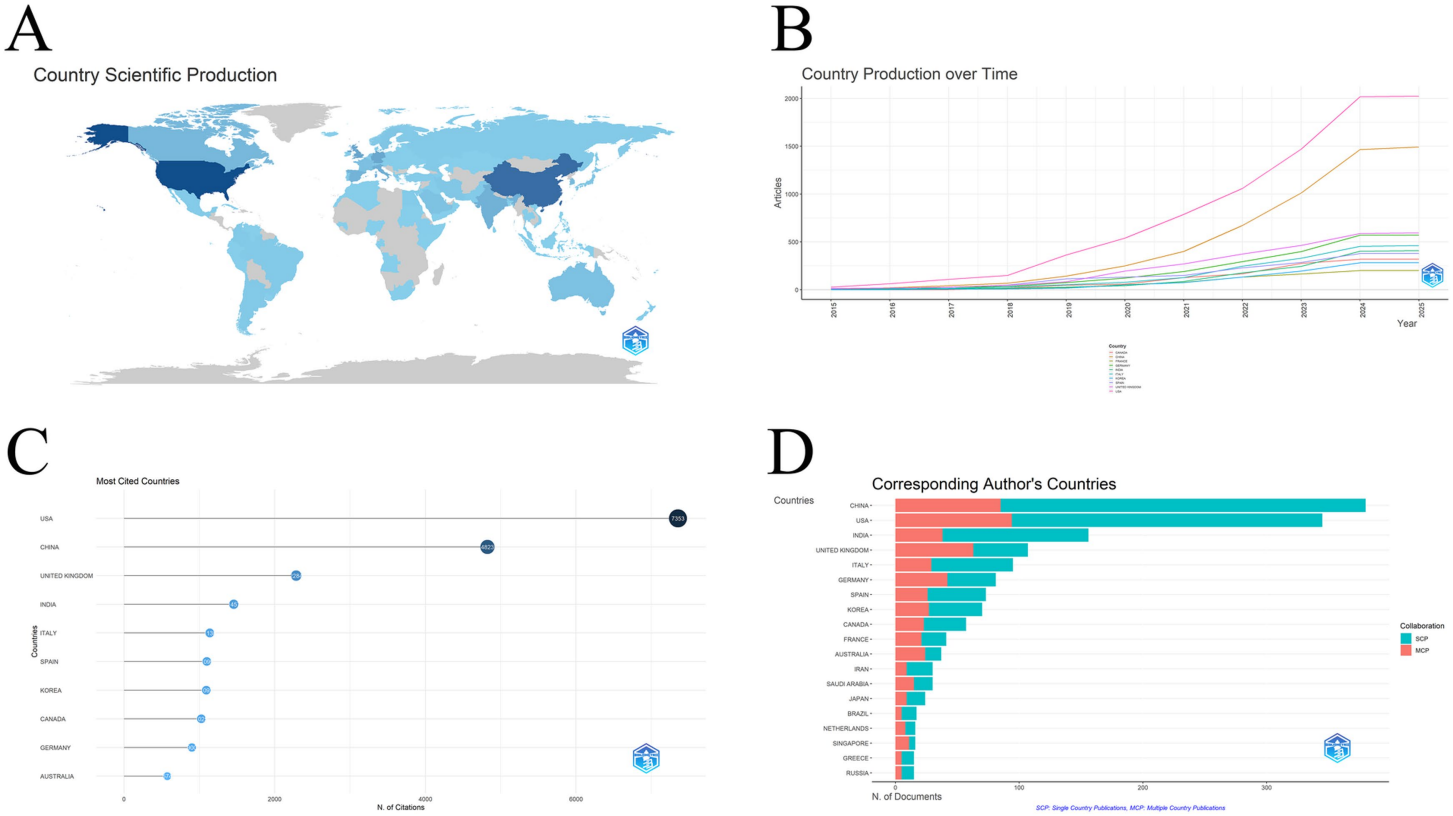
**(A)** An overview of the number of target publications in each country or region, where darker colors mean more publications; **(B)** The top 10 countries or regions in terms of the number of articles sent over time. The figure shows that there is a clear gap between China and the United States compared to other countries; **(C)** The top 10 countries or regions by citation frequency reflect the influence of each country or region in this field; **(D)** The number of authors by country and region and proportion of international cooperation. The figure reflects the number of corresponding authors from each country and their ability to collaborate internationally.

Analyzing from the perspective of countries, the general situation of each country and region can be seen in Figure 7A, where the color shades reflect the number of articles issued, and it is obvious that the U.S. plate is the darkest, which means that it has the largest number of articles issued, followed by China. Analyzing the graph of year-by-year output of the top 10 countries or regions in terms of the number of articles issued (Figure 7B), it is clear to observe the leading position of the United States during this decade, and China’s year-by-year output is not low relative to the remaining eight countries. The major research in this field over the decade has been conducted by both the United States and China. The U.S. leads not only in terms of the number of publications, but also in the number of citations, with 7,353, which is more than one and a half times that of China in second place. China is second with 4,823, and third is the UK with 2,284 citations (Figure 7C). China and the U.S. lead the other countries in a cliff, which shows the low position of China and the U.S. as leaders in this field. Figure 7D shows the authors and international collaborations by country. Authors from China are the largest, with 85 co-authors with foreign authors, 295 domestic co-authors only, and a total of 380 publications, followed by the United States, with 94 authors with international collaborations, 251 domestic co-authors only, and a total of 345 publications. Details of the remaining eight countries or regions are in Table 4.

**Table 4:** The top 10 countries of authors and international cooperation.

Country	Articles	SCP	MCP	Freq	MCP_Ratio
CHINA	380	295	85	0.198	0.224
USA	345	251	94	0.18	0.272
INDIA	156	118	38	0.081	0.244
UNITED KINGDOM	107	44	63	0.056	0.589
ITALY	95	66	29	0.049	0.305
GERMANY	81	39	42	0.042	0.519
SPAIN	73	47	26	0.038	0.356
KOREA	70	43	27	0.036	0.386
CANADA	57	34	23	0.03	0.404
FRANCE	41	20	21	0.021	0.512

### 3.7 Document and references

**Figure 8:**
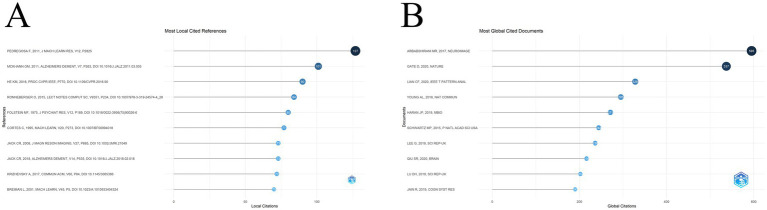
**(A)** The figure shows the 10 most cited articles in the target literature, reflecting their significant impact on the field; **(B)** The top 10 globally cited target literature reflects their global reach.

Evaluating the cited literature reveals studies that are relatively important to this field in terms of previous research. At the top of the list of most locally cited references (Figure 8A) is an article by F. Pedregosa, G. Varoquaux, et al. It has been cited a total of 127 times. This article describes Scikit-learn, which provides a wide range of machine learning tools for Python, greatly contributing to the popularity of Python in the field of machine learning and lowering the barriers to implementing machine learning algorithms (Pedregosa et al., 2011). Second on the list is an article by Mckhann GM, D. S. Knopman et al. on diagnostic criteria for Alzheimer’s disease. The article emphasized on early diagnostic criteria. They emphasized the importance of early diagnosis and proposed some new clinical, imaging and biomarker diagnostic criteria (McKhann et al., 2011). The third paper is about Resnet model by Kaiming He, Xiangyu Zhang and others. This model solves the problem of gradient vanishing and degradation in the training of traditional deep neural networks by introducing “residual connection,” which enables the successful training of very deep networks (He et al., 2016). The remaining seven articles are shown in Table 5. Table 6 shows the outbreak citations in the last decade, and four papers have been used since 2023, one of which is a review on Alzheimer’s disease by Schelten P et al. published in The Lancet in 2021, which reviews the latest research progress in Alzheimer’s disease, including its pathomechanisms, clinical manifestations, and so on (Scheltens et al., 2021). In addition, there are two review articles on Parkinson’s, published by two teams represented by Tolosa E and Bloem BR (Tolosa et al., 2021; Bloem et al., 2021). The last one, published in 2020 and authored by Livingston G et al. published in The Lancet, this report summarizes the latest research advances in dementia prevention, intervention, and care, suggests nine possible risk factors for repair, and emphasizes the importance of multidisciplinary collaboration in the management of dementia (Livingston et al., 2020). In Figure 8B is a ranking of the top 10 most cited articles over the decade. The three highly cited articles are: a review by Arbabshirani et al. (2017) published in NeuroImage, which explores recent advances in neuroimaging-based prediction of brain disorders in a single subject, with a focus on the use of structural, functional, and diffusion MRI in classification studies of psychiatric and neurodegenerative disorders such as schizophrenia and Alzheimer’s disease, using structural, functional and diffusion MRI and other neuroimaging methods with machine learning techniques (Arbabshirani et al., 2017). Its outstanding performance of 595 total citations (66.11 per year) reflects the importance of this review to the field. Gate et al. (2020) published “Clonally expanded CD8 T cells patrol the cerebrospinal fluid of patients with Alzheimer’s disease” in Nature, revealing the critical role of CD8 + T_EMRA cells in disease immunomodulation, with a total of 537 citations (89.5 per year) and a high standardized citation index of 16.33, highlighting its potential to open up new pathways for targeted therapy through pathological innovations. With 537 total citations (89.5 per year) and a high standardized citation index of 16.33, it highlights that it opens up possible new pathways for targeted therapies through innovations in pathomechanisms (Gate et al., 2020). Lian et al. (2018) proposed “Hierarchical Fully Convolutional Network” (H-FCN) in IEEE T PATTERN ANAL, which utilizes deep learning to jointly analyze the localization of cerebral atrophy and the structural information of the whole brain, to improve the diagnostic accuracy of Alzheimer’s disease, with a total of 328 citations (annual average of 54.67), confirming the value of AI-driven medical image analysis technology. The total number of citations is 328 (annual average 54.67), which confirms the technical value of AI-driven medical image analysis (Lian et al., 2018). More information is presented in Table 7.

**Table 5:** The top 10 references cited locally.

Cited references	Citations
PEDREGOSA F, 2011, J MACH LEARN RES, V12, P2825	127
MCKHANN GM, 2011, ALZHEIMERS DEMENT, V7, P263, DOI 10.1016/J.JALZ.2011.03.005	101
HE KM, 2016, PROC CVPR IEEE, P770, DOI 10.1109/CVPR.2016.90	90
RONNEBERGER O, 2015, LECT NOTES COMPUT SC, V9351, P234, DOI 10.1007/978-3-319-24574-4_28	84
FOLSTEIN MF, 1975, J PSYCHIAT RES, V12, P189, DOI 10.1016/0022-3956(75)90026-6	80
CORTES C, 1995, MACH LEARN, V20, P273, DOI 10.1007/BF00994018	77
JACK CR, 2008, J MAGN RESON IMAGING, V27, P685, DOI 10.1002/JMRI.21049	73
JACK CR, 2018, ALZHEIMERS DEMENT, V14, P535, DOI 10.1016/J.JALZ.2018.02.018	73
KRIZHEVSKY A, 2017, COMMUN ACM, V60, P84, DOI 10.1145/3065386	72
BREIMAN L, 2001, MACH LEARN, V45, P5, DOI 10.1023/A:1010933404324	70

**Table 6:** Top 25 references with the strongest citation bursts.

References	Year	Strength	Begin	End	2015–2025
Liu SQ, 2015, IEEE T BIO-MED ENG, V62, P1132, DOI 10.1109/TBME.2014.2372011, DOI	2015	7.89	**2017**	2020	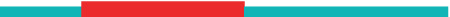
Moradi E, 2015, NEUROIMAGE, V104, P398, DOI 10.1016/j.neuroimage.2014.10.002, DOI	2015	5.91	**2017**	2020	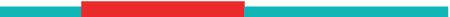
Salvatore C, 2014, J NEUROSCI METH, V222, P230, DOI 10.1016/j.jneumeth.2013.11.016, DOI	2014	5.81	**2017**	2019	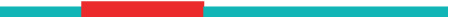
Bron EE, 2015, NEUROIMAGE, V111, P562, DOI 10.1016/j.neuroimage.2015.01.048, DOI	2015	4.92	**2017**	2020	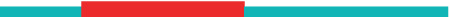
He KM, 2016, PROC CVPR IEEE, V0, PP770, DOI 10.1109/CVPR.2016.90, DOI	2016	10.09	**2018**	2021	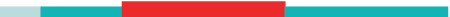
Srivastava N, 2014, J MACH LEARN RES, V15, P1929	2014	6.08	**2018**	2019	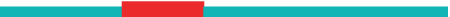
Goodfellow I, 2016, ADAPT COMPUT MACH LE, V0, P1	2016	5.63	**2018**	2021	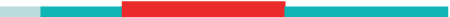
Ortiz A, 2016, INT J NEURAL SYST, V26, P0, DOI 10.1142/S0129065716500258, DOI	2016	5.23	**2018**	2021	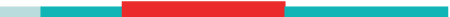
Ronneberger O, 2015, LECT NOTES COMPUT SC, V9351, P234, DOI 10.1007/978-3-319-24574-4, 28, DOI	2015	7.76	**2019**	2020	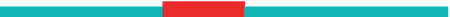
Litjens G, 2017, MED IMAGE ANAL, V42, P60, DOI 10.1016/j.media.2017.07.005, DOI	2017	7.44	**2019**	2021	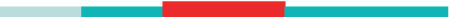
Postuma RB, 2015, MOVEMENT DISORD, V30, P1591, DOI 10.1002/mds.26424, DOI	2015	5.53	**2019**	2020	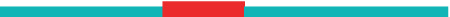
Shen DG, 2017, ANNU REV BIOMED ENG, V19, P221, DOI 10.1146/annurev-bioeng-071516044442, DOI	2017	5.53	**2019**	2020	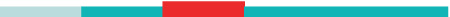
Rusk N, 2016, NAT METHODS, V13, P35, DOI 10.1038/nmeth.3707, DOI	2016	4.98	**2019**	2020	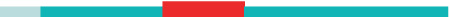
Cole JH, 2017, NEUROIMAGE, V163, P115, DOI 10.1016/j.neuroimage.2017.07.059, DOI	2017	4.81	**2019**	2022	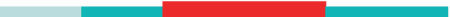
Krizhevsky A, 2017, COMMUN ACM, V60, P84, DOI 10.1145/3065386, DOI	2017	9.18	**2020**	2022	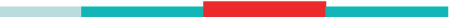
Gaugler J, 2019, ALZHEIMERS DEMENT, V15, P321, DOI 10.1016/j.jalz.2019.01.010, DOI	2019	5.52	**2020**	2022	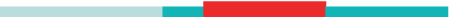
Poewe W, 2017, NAT REV DIS PRIMERS, V3, P0, DOI 10.1038/nrdp.2017.13, DOI	2017	4.99	**2020**	2022	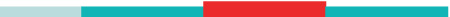
Vieira S, 2017, NEUROSCI BIOBEHAV R, V74, P58, DOI 10.1016/j.neubiorev.2017.01.002, DOI	2017	4.89	**2020**	2021	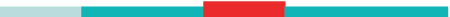
Rathore S, 2017, NEUROIMAGE, V155, P530, DOI 10.1016/j.neuroimage.2017.03.057, DOI	2017	6.73	**2021**	2022	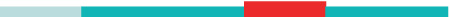
Wang SH, 2018, J MED SYST, V42, P0, DOI 10.1007/s10916-018-0932-7, DOI	2018	4.91	**2021**	2022	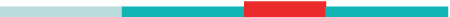
Dorsey ER, 2018, LANCET NEUROL, V17, P939, DOI 10.1016/S1474-4422(18)30295-3, DOI	2018	4.65	**2022**	2023	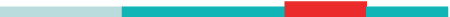
Scheltens P, 2021, LANCET, V397, P1577, DOI 10.1016/S0140-6736(20)32205-4, DOI	2021	6.5	**2023**	2025	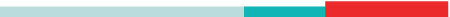
Tolosa E, 2021, LANCET NEUROL, V20, P385, DOI 10.1016/S1474-4422(21)00030-2, DOI	2021	5.68	**2023**	2025	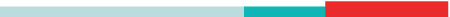
Bloem BR, 2021, LANCET, V397, P2284, DOI 10.1016/S0140-6736(21)00218-X, DOI	2021	4.86	**2023**	2025	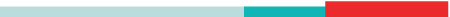
Livingston G, 2020, LANCET, V396, P413, DOI 10.1016/S0140-6736(20)30367-6, DOI	2020	4.86	**2023**	2025	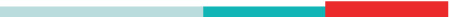

Bold values mean the beginning of the strongest citation burst.

**Table 7:** The top 10 cited articles in this field for a decade.

Paper	DOI	Total citations	TC per year	Normalized TC
ARBABSHIRANI MR, 2017, NEUROIMAGE	10.1016/j.neuroimage.2016.02.079	595	66.11	10.43
GATE D, 2020, NATURE	10.1038/s41586-019-1895-7	537	89.50	16.33
LIAN CF, 2020, IEEE T PATTERN ANAL	10.1109/TPAMI.2018.2889096	328	54.67	9.97
YOUNG AL, 2018, NAT COMMUN	10.1038/s41467-018-05892-0	295	36.88	6.38
HARAN JP, 2019, MBIO	10.1128/mBio.00632-19	271	38.71	5.65
SCHWARTZ MP, 2015, P NATL ACAD SCI USA	10.1073/pnas.1516645112	244	22.18	6.41
LEE G, 2019, SCI REP-UK	10.1038/s41598-018-37769-z	236	33.71	4.92
QIU SR, 2020, BRAIN	10.1093/brain/awaa137	216	36.00	6.57
LU DH, 2018, SCI REP-UK	10.1038/s41598-018-22871-z	202	25.25	4.37
JAIN R, 2019, COGN SYST RES	10.1016/j.cogsys.2018.12.015	190	27.14	3.96

## 4 Discussion

### 4.1 Global context

With the aging of the population, the number of patients with neurodegenerative diseases will gradually increase. In recent years, AI technology has made a lot of progress globally, with varying degrees of application in various fields, showing diverse trends. In the medical field, the application of AI is becoming more and more in-depth, such as the use of machine learning medical image recognition, analyzing medical data and forecasting, discovering drug targets and screening compounds. In the field of neurodegenerative diseases, researchers and organizations worldwide are working to explore key pathogenic pathways and potential drug targets using AI technology. Not only that, the combination of AI and brain-computer interface shows great potential in the field of neurorehabilitation. With the aid of AI technology, brain-computer interfaces can be personalized according to the patient’s neural signals, which is conducive to the designation of a more accurate rehabilitation program, and patients with neurodegenerative diseases are expected to regain some of their self-care ability.

### 4.2 Knowledge map

The volume of literature continues to grow from 2015 to 2025, which is highly synchronized with the maturation of AI technology, especially after 2019. This trend reflects the important role of AI technologies in the early diagnosis of neurodegenerative diseases (e.g., MRI analysis of Alzheimer’s disease) and in the analysis of pathomechanisms.

As a comprehensive open access journal, *Scientific Reports* (a division of *Nature Publishing Group*) aims to “cover a wide range of natural sciences and clinical medicine,” with particular emphasis on the rapid dissemination of interdisciplinary research. Its review process balances innovation and technical rigor, and although its impact factor is moderate (IF = 4.6 in 2023), it has become one of the preferred platforms for early-stage exploratory AI healthcare research due to its open access policy and “Nature” brand endorsement. *Frontiers in Aging Neuroscience* (*Frontiers Press*), on the other hand, focuses on the molecular mechanisms and intervention strategies of aging and neurodegenerative diseases, integrating basic research and clinical translational results in a topical format, with an IF = 5.8 in 2023, and ranked in the neuroscience segment in Q1. It’s fast-paced publication model that attracts a large number of time-sensitive AI Pathogenesis Research. *Neuroimage* (published by *Elsevier*), as a top journal in neuroimaging (IF = 6.4 in 2023), has long been dedicated to imaging technology development and brain disease applications, and is demanding on methodological innovations, and its high rejection rate ensures that its articles are cutting-edge and technologically in-depth. *IEEE Access* (*IEEE Press*), although a multidisciplinary engineering journal, has been favored by AI algorithmic research in recent years due to its open access and fast review (IF = 3.9 in 2023). Of note, the *Journal of Alzheimer’s Disease* (*IOS Press*), as a specialty journal (IF = 4.0 in 2023), features a full chain of research “from molecule to clinic,” providing a vertical publication channel for AI-driven biomarker AI-driven biomarker discovery with a vertical publication channel. Together, the differentiated positioning of these journals forms a multifaceted knowledge ecosystem for research at the intersection of neurodegenerative diseases and AI.

At the intersection of neurodegenerative diseases and AI, the long-term investment and interdisciplinary layout of some institutions have laid the foundation for their academic impact. The University of California System, as the world’s largest public research university consortium, has established a number of interdisciplinary research centers (e.g., UCSF Memory and Aging Center) through the integration of neuroscience, computer science and clinical medicine resources, and is committed to promoting AI in early biomarkers of neurodegenerative diseases. For example, its team developed a deep learning-based framework for cerebrospinal fluid proteome analysis, which provides a new method for classifying Alzheimer’s disease subtypes (Tang et al., 2019). Relying on the clinical resources of the National Hospital for Neurology and Neurosurgery (NHNN) and the machine learning strengths of the Department of Computing, University College London (UCL) focuses on the fusion analysis of multimodal data (e.g., PET-MRI images and genetic data), and its development of open-source toolkits (e.g., ANTs, FSLs) have become one of the standard processes for brain image processing (Iglesias, 2023; Billot et al., 2020). The University of Chinese Academy of Sciences (UCAS), on the other hand, has made breakthroughs in the development of AI models for gait analysis in Parkinson’s disease through synergistic innovation between engineering and clinical research, and the relevant algorithms have already been applied to data parsing in wearable devices (Chen et al., 2020). The commonality of these institutions lies in the fact that through infrastructure sharing, interdisciplinary team establishment and industry-academia-research linkage, the barriers to technological translation are systematically lowered, thus creating sustainable research outputs in the niche areas.

Benzinger Tammie, highlighted in the Collaborative Network, is a researcher from Washington University. She was named a Highly Cited Researcher in Crosscutting Areas in 2020–2022 and a Highly Cited in Neuroscience and Behavior in 2023–2024. Researcher. According to WoS, she has a total of 303 articles with 15,859 citations and an H_index of 57 from 2015, but she does not appear in the top eight H_index list for this study, suggesting that this may not be her area of expertise. In 2019, she and her team investigated cerebral blood–brain barrier damage using novel cerebrospinal fluid biomarkers and dynamic contrast-enhanced magnetic resonance imaging, and found that blood–brain barrier disruption is an early biomarker of cognitive dysfunction in humans independent of Aβ and tau (Nation et al., 2019). In 2020, she and her team went on to discover that variants in APOE4 are a major susceptibility gene for Alzheimer’s disease, and that individuals carrying APOE4 are distinguished from those without by breakdown of the blood–brain barrier in the hippocampus and medial temporal lobe. Studies have shown that disruption of the blood–brain barrier contributes to APOE4-associated cognitive decline, which may be a therapeutic target for Alzheimer’s disease (Montagne et al., 2020). Topping the list of local citations is Finger Elizabeth, with only two articles in the selected time interval, but the highest ranking in local citations, which shows the extraordinary influence of this author in the field. Of the two articles, one describes a machine learning technique called subtype and stage inference, which provides fine-grained patient stratification and improves the ability to predict between diagnostic categories (Young et al., 2018). The other article is about the development of a comprehensive framework utilizing multimodal recurrent neural networks with the integration of longitudinal multidomain data. Using this framework, the accuracy of predictive models for MCI to AD conversion was improved (Lee et al., 2019). The second most locally cited researcher is Shen Dinggang from Shanghai Tech University, who has been continuously recognized as a Highly Cited Researcher in Crosscutting Areas from 2021 to 2024. His most highly cited article is a review published in 2017 that reviews the application of computer-aided image analysis in medical imaging and advances in machine learning prior to 2017, and suggests directions for improvement (Shen et al., 2017). Another study published in 2011 suggests a recommendation to change the traditional single biomarker diagnostic paradigm to one that combines three biomarkers in a nuclear combination approach to differentiate between Alzheimer’s disease and healthy individuals. Controlled experiments were conducted to validate the accuracy of this approach (Zhang et al., 2011).

In terms of countries, the United States is the main contributor to the field, with the highest number of publications and citation frequency. China follows, with both countries leading the rest of the world, showing that the U.S. and China are world leaders in the field of AI. According to the current situation, the United States and China will remain in the lead for a long time to come. It can even be said that the competition in the field of AI is only with the United States and China. For this reason, we have analyzed the possible driving forces behind the two countries.

China and the United States are both countries with aging populations, and as the population ages, many social problems are emerging. In the medical field, the focus is on the composition and proportion of diseases in society. The aging of the population also implies an “aging” of the types of diseases, with geriatric diseases, such as neurodegenerative diseases, slowly taking over. In response to this evolution, research on neurodegenerative diseases has increased in China and the United States: the National Institutes of Health (NIH), the main funding agency in the United States, continues to invest heavily in neurodegenerative disease research, including the use of AI. Non-governmental organizations (NGOs) are also actively investing in research, such as the Milken Institute and the Biswas Family Foundation, which have invested nearly $15 million in the Transformative Computational Biology Grants Program, which focuses on the use of AI in neurodegenerative diseases. In China, the Chinese government has led the “Alarm Program,” which places a strategic focus on AI and brain science research. Hong Kong’s InnoHK Innovation Cluster Initiative provided an initial HK$500 million grant to the Hong Kong Center for Neurodegenerative Diseases (HKCeND), which was established by the Hong Kong University of Science and Technology with the aim of creating a world-leading research center. Not only that, tech giants such as Tencent have also started investing in the healthcare sector. The influx of investment has allowed researchers to have sufficient resources to conduct various studies. The U.S. has been a leader in AI due to its early AI research, and they have used this advantage to apply to medicine and continue to carry out research to establish its position as a global leader. Although China started later, AI has been developing rapidly in recent years, and has even achieved unique advantages and potential in certain aspects, such as medical image analysis, disease risk prediction and public health, and the intelligence of Chinese medicine. Although other countries have also invested in this field, the scale of funding, strategic importance, data resources and other aspects are not as good as China and the United States, which has also established their backward position.

### 4.3 Applications of AI in neurodegenerative diseases

Based on the cluster analysis of keywords, we identified seven major research topic clusters. This reflects the diversity of research hotspots in this field.

#### 4.3.1 Immune infiltration

Immune infiltration, which refers to the process by which immune cells enter the nervous system, plays an important role in a variety of neurodegenerative diseases. AI technologies, particularly deep learning algorithms, have been used to analyze neuroimaging data to identify the distribution and activity of immune cells (Li et al., 2022; Liu et al., 2021).

#### 4.3.2 Parkinsons disease

AI has important applications in the early diagnosis of Parkinson’s disease and the study of disease mechanisms. For example, an automatic Parkinson’s disease detection system based on convolutional neural network can analyze the abnormal signals of human electroencephalogram, which brings a new way for early diagnosis of Parkinson’s disease (Chen et al., 2020; Oh et al., 2020). In addition, AI is also used in wearable devices to collect and analyze users’ daily health data for early diagnosis and personalized treatment.

#### 4.3.3 Multiple sclerosis

AI shows great potential in the diagnosis and lesion assessment of multiple sclerosis. It has been shown that MS lesions can be segmented based on a variety of neural network models. Not only that, AI also plays an important role in the identification of drug targets (Brosch et al., 2016; Kamnitsas et al., 2017; Myszczynska et al., 2020). In addition, AI is also used in wearable devices to collect and analyze users’ daily health data for early diagnosis and personalized treatment (Zeng et al., 2020).

#### 4.3.4 Mild cognitive impairment

Mild Cognitive Impairment (MCI) mediates the transition between age-related cognitive decline and Alzheimer’s Disease. AI technologies have important applications in the identification and risk prediction during MCI. Machine learning algorithms are able to integrate multiple data sources such as neuroimaging, biomarkers, and clinical assessments to provide more accurate predictive models of MCI (Moradi et al., 2015; Ding et al., 2019).

#### 4.3.5 Deep learning

Deep learning is used in a wide range of neurodegenerative diseases, including automated analysis of brain imaging data to identify early signs of disease, improve diagnostic efficiency and accuracy as well as predict disease progression and identify drug targets (Lian et al., 2018; Lee et al., 2019; Vásquez-Correa et al., 2018; Feng et al., 2019).

#### 4.3.6 Machine learning

Machine learning plays a key role in diagnostic and therapeutic decisions for neurodegenerative diseases. By analyzing multi-omics data, machine learning algorithms are able to identify potential biomarkers of disease and construct predictive models (Cha et al., 2024; Dennis and Strafella, 2024).

#### 4.3.7 Freesurfer

Freesurfer is a tool for neuroimaging analysis that is widely used for automated analysis of brain structures. In the study of neurodegenerative diseases, Freesurfer is used to analyze brain imaging data and identify disease-related structural changes (Pizzini et al., 2024; Ahmad et al., 2024; De Bonis et al., 2024).

#### 4.3.8 Scale

In the study of neurodegenerative diseases, “scale” refers to the method of analyzing data at different scales, and AI technology is used to analyze data at multiple scales, from the molecular level to the tissue level, to help researchers understand the mechanisms of the disease in a comprehensive way (Adibi et al., 2025; Bevilacqua et al., 2025).

### 4.4 Research trends

Based on our detailed analysis of the bibliometrix and CiteSpace maps, we hypothesize that the following four research trends or hotspots are likely to emerge in the near future.

#### 4.4.1 Accurate diagnosis of multimodal data fusion

Based on the continued activity of “diagnosis,” “deep learning” and “mr-images” in the trending topics, combined with the long-term continuation of “classification” and “segmentation” in the keyword clustering, we hypothesize that AI will further promote multimodal data (e.g., brain data) in the future. Based on the continued activity of “classification” and “segmentation” in the trending topics, combined with the long-term continuation of “classification” and “segmentation” in the keyword clustering, we hypothesize that AI will further promote the deep fusion of multimodal data (e.g., brain images, genome, clinical indicators to solve neurodegenerative problems in the future, clinical indicators) deep fusion to address heterogeneous diagnosis of neurodegenerative diseases. Examples such as early diagnosis or prediction of neurodegenerative diseases through various diagnostic modalities such as radiogenomics or facial expression analysis (Rao et al., 2025; Goudarzi et al., 2025).

#### 4.4.2 Case mechanism analysis and target discovery

The rising trend of the keywords “docking” and “pathogenesis” combined with the prominence of immune mechanisms and protein aggregation studies in the highly cited literature suggests that AI will accelerate the exploration of molecular mechanisms. For example, using AI to predict certain protein denaturation in neurodegenerative diseases or to monitor the effects of certain natural compounds (Gonay et al., 2025; Tripathi and Kumar, 2025).

#### 4.4.3 Interpretability and clinical translation

The persistence of the keywords “risk” and “disease progression” (2015–2024), and the long-term activity of “diagnosis,” reflect the clinical awareness of the dynamic prognosis of disease. Long-term activity, reflecting the clinical need for dynamic prognostic modeling. Interpretable AI (XAI) tools (e.g., SHAP) can enhance physicians’ trust in AI decisions by visualizing feature contributions. Examples include multimodal variables for predicting the progression of Parkinson’s disease, the PIDGN model for early prediction of Parkinson’s disease and the ChiGa-Net neural network for diagnosis and monitoring of Parkinson’s disease (Jiang et al., 2024; Li et al., 2025; Ali et al., 2025).

#### 4.4.4 Technical differentiation of segmented diseases

The continuation of the keyword “parkinsons disease” (2015–2024) and the emerging trend of “huntingtons-disease” (emerging after 2021) suggests that AI needs to Develop specialized technologies for disease-specific needs. For example, the quantification of motor symptoms in Parkinson’s disease relies on high-frequency sensor data, while Huntington’s disease requires a focus on CAG repeat sequence modulation.

## 5 Limitations

This study uses bibliometric methods to analyze the existing literature, to mine and present the main information under the field, and to speculate on possible research trends. However, bibliometric analysis cannot provide specific treatment options or diagnostics, but only serves as a visual knowledge graph for researchers to understand. And although the prediction of future research trends strictly adheres to the latest known research, there is still a small probability of bias. The current study is also time-sensitive because the collection of the target literature was cut off at the beginning of the study, and subsequent literature could not be included in the analysis.

## 6 Conclusion

In this study, a total of 1,956 relevant articles in this field were collected for bibliometric analysis over the period 2015–2025, identifying the major contributing journals, institutions, authors and countries. Some of the important studies by the main contributing authors, information about the institutions and journals and the funding and policy situation behind the countries were explored. The results of keyword-centered clustering of this literature are analyzed, and the results are used to illustrate the current state of the art in the use of AI in neurodegenerative diseases and to predict possible subsequent research trends. Since AI is still in a rapid development stage, we recommend that researchers in this field utilize AI to develop more tools to meet the challenges posed by complex disease diagnosis and treatment systems and socio-demographic changes, and accelerate the movement of AI into the clinic to meet clinical needs.

## Data Availability

The raw data supporting the conclusions of this article will be made available by the authors without undue reservation.
